# Supplementary material for: Caries risk assessment by Caries Management by Risk Assessment (CAMBRA) Protocol among the general population of Pakistan–a multicenter analytical study
Source: PeerJ. 2024 Jan 31;12:e16863. doi: 10.7717/peerj.16863 (PMC10838085; doi:10.7717/peerj.16863)
Supplement: Supplemental Information 2 [file peerj-12-16863-s002.doc]

**Completed STROBE checklist**

This checklist was elaborated using formal items recommended for cross-sectional studies from STROBE statement (https://www.strobe-statement.org).

|  | Item No | Recommendation | Respected ? | Comments and quotes | |
| --- | --- | --- | --- | --- | --- |
| **Title and abstract** | 1 | (*a*) Indicate the study’s design with a commonly used term in the title or the abstract | Yes | | Study design is indicated in the Methods section of the abstract  “This was a multicenter analytical study” |
|  |  | (*b*) Provide in the abstract an informative and balanced summary of what was done and what was found | Yes | | This information are stated in the study abstract (study objective described, method and results described) |
| Introduction | | |  | |  |
| Background/rationale | 2 | Explain the scientific background and rationale for the investigation being reported | Yes | | Rationale and existing literature are stated in the introduction section |
| Objectives | 3 | State specific objectives, including any prespecified hypotheses | Yes | | A statement at the end of the introduction specifies the specific goals and objectives.  “The aim of this study was to assess the caries risk using Caries Management by Risk Assessment (CAMBRA) protocol among the general population of Pakistan. |
| Methods | | |  | |  |
| Study design | 4 | Present key elements of study design early in the paper | Yes | | Study design is stated in the first subsection of Methods. Key elements are all described in the methods.  This was a multicenter analytical study |
| Setting | 5 | Describe the setting, locations, and relevant dates, including periods of recruitment, exposure, follow-up, and data collection | Mostly | | Setting, contexts, dates of inclusion, are fully described in the method section .  This multicenter analytical study was carried out in ten dental hospitals of different provinces of Pakistan (Punjab, Sindh, KPK). |
| Participants | 6 | (*a*) Give the eligibility criteria, and the sources and methods of selection of participants | Yes | | Study population is described is the method section, as well as selection criteria  “The participants were included if they were above the age of 6 years, understood English, Hindko, Pushto, Punjabi, and Urdu language, and were residents of Pakistan.” |
| Variables | 7 | Clearly define all outcomes, exposures, predictors, potential confounders, and effect modifiers. Give diagnostic criteria, if applicable | Yes | | Standardized variable definitions were used across all programs, which are presented in method section. |
| Data sources/ measurement | 8* | For each variable of interest, give sources of data and details of methods of assessment (measurement). Describe comparability of assessment methods if there is more than one group | Yes | | Data collection and measurement was the same for all variables, and is described in the methods section.  The socio-demographic information was collected which included age, gender, education, profession, and socio-economic status. The caries risk assessment was carried out using a questionnaire which was designed using the CAMBRA caries risk assessment tool which is a practical and predictable tool that includes almost all the factors related to the disease. The CAMBRA tool comprises of eight risk and protective factors and four disease indicators. |
| Bias | 9 | Describe any efforts to address potential sources of bias | Yes | | We notably tried to reduce bias by excluding incomplete questionnaire. The analysis section also explains this part. |
| Study size | 10 | Explain how the study size was arrived at | Yes | | The method describes the sample size estimation for this study. |
| Quantitative variables | 11 | Explain how quantitative variables were handled in the analyses. If applicable, describe which groupings were chosen and why | Yes | | Definitions of all categories for variables are presented method section.  The socio-demographic variables were collected which included age, gender, education, profession, and socio-economic status. The caries risk assessment was carried out using a questionnaire which was designed using the CAMBRA caries risk assessment tool which is a practical and predictable tool that includes almost all the factors related to the disease. |
| Statistical methods | 12 | (*a*) Describe all statistical methods, including those used to control for confounding | Yes | | The data was analysed using Statistical Package for the Social Sciences (SPSS IBM, Chicago, IL, USA) version 27. The percentages, frequency, mean, and standard deviation were obtained in the descriptive analysis. To find the significant factors associated with caries (i.e., moderate, high, and extremely high) logistic regression test was carried out. Simple logistic regression analysis was initially performed to obtain the crude odds ratio (COR), and those that had a p-value of less than 0.25 were considered important predictors of caries and included in the multiple logistic regression analysis to obtain their adjusted odds ratio (AOR). Variable were selected using forward LR and Back LR methods, and Enter method was run to obtain the final model. |
|  |  | (*b*) Describe any methods used to examine subgroups and interactions | Yes | | This is described in the method section. The participants were asked to fill in that questionnaire, and they went through intra-oral clinical examination, and a bitewing radiograph were taken for their oral hygiene status evaluation and detection of any caries lesions. |
|  |  | (*c*) Explain how missing data were addressed | Yes | | This is described in the method section.  “Missing values were not inferred” |
|  |  | (*d*) If applicable, describe analytical methods taking account of sampling strategy | N/A | | Non applicable |
|  |  | (*e*) Describe any sensitivity analyses | N/A | | Non applicable |
| Results | | |  | |  |
| Participants | 13* | (a) Report numbers of individuals at each stage of study—eg numbers potentially eligible, examined for eligibility, confirmed eligible, included in the study, completing follow-up, and analysed | Yes | | This is described at the beginning of result section.  “The study involved 521 patients and higher number of participants (61.2%) were found to be in the moderate risk category and only 13.4%, were in the ‘low’ category of caries risk assessment” |
|  |  | (b) Give reasons for non-participation at each stage | Yes | | This is described at the beginning of result section.  The study involved 521 patients and higher number of participants (61.2%) were found to be in the moderate risk category and only 13.4%, were in the ‘low’ category of caries risk assessment. |
|  |  | (c) Consider use of a flow diagram | N/A | | Use of a flow diagram was not deemed appropriate. |
| Descriptive data | 14* | (a) Give characteristics of study participants (eg demographic, clinical, social) and information on exposures and potential confounders | Yes | | Table 1 describes the participants and programs included.  “Table 1: Socio-demographic characteristics of the participants. |
|  |  | (b) Indicate number of participants with missing data for each variable of interest | Yes | | The total numbers of recorded data for each variable are stated in variable headline of each table |
| Outcome data | 15* | Report numbers of outcome events or summary measures | Yes | | All numbers are reported in Tables. |
| Main results | 16 | (*a*) Give unadjusted estimates and, if applicable, confounder-adjusted estimates and their precision (eg, 95% confidence interval). Make clear which confounders were adjusted for and why they were included | N/A | | N/A |
|  |  | (*b*) Report category boundaries when continuous variables were categorized | Yes | | Category boundaries are displayed in variable headings in the tables, where applicable (age groups) |
|  |  | (*c*) If relevant, consider translating estimates of relative risk into absolute risk for a meaningful time period | N/A | | N/A |
| Other analyses | 17 | Report other analyses done—eg analyses of subgroups and interactions, and sensitivity analyses | N/A | | N/A |
| Discussion | | |  | |  |
| Key results | 18 | Summarise key results with reference to study objectives | Yes | | Key results are described at the beginning of discussion section (page 20), and later on by the mean of a paragraph displaying main operational implications. They also are summarized in the conclusion |
| Limitations | 19 | Discuss limitations of the study, taking into account sources of potential bias or imprecision. Discuss both direction and magnitude of any potential bias | Yes | | Description of limitations is done at the end of discussion section. |
| Interpretation | 20 | Give a cautious overall interpretation of results considering objectives, limitations, multiplicity of analyses, results from similar studies, and other relevant evidence | Yes | | References were added where possible and discussed. Limitations were taken into account in the discussion. |
| Generalisability | 21 | Discuss the generalisability (external validity) of the study results | Yes | | Study generalizable described in the discussion section. |
| Other information | | |  | |  |
| Funding | 22 | Give the source of funding and the role of the funders for the present study and, if applicable, for the original study on which the present article is based | Yes | | Funding information were displayed upon submission but not included in the manuscript, as requested. |
|  | | |  | |  |

*Give information separately for exposed and unexposed groups.
